# Minutes of the Regular Annual Meeting of the West Texas Medical Association

**Published:** 1886-12

**Authors:** D. Berry

**Affiliations:** San Antonio, Texas


### MINUTES OF THE REGULAR ANNUAL MEETING OF THE WEST TEXAS MEDICAL ASSOCIATION,

HELD OCTOBER 27, 1886.

*By D. Berry, M. D., San Antonio, Texas.*

(For Daniel's Texas Medical Journal).

Regular annual meeting of West Texas Medical Association called to order at 8 p. m., October 27, 1886, the President, Dr. G. Graham Watts, in the chair. Present, Drs. Cupples, Chew, Tyner, Tyler, Lowry, Barnitz, Carothers, Terrell, Menger, Favre, Fennell of Seguin, Kingsley, King and Berry.

The minutes of the last annual meeting were read by the secretary. The annual report of the treasurer was read and adopted. The secretary read a telegram from Dr. G. W. Kerr, of Waelder, regretting his absence. Dr. Tyner presented the name of Dr. T. G. Turpin, of Corpus Christi, for membership. It was determined by the censors to lay the matter over until the next regular meeting in order to obtain the gentleman's credentials. No other business appearing before the Association for disposal, the election of officers for the ensuing year was proceeded with. For President the names of Drs. Lowry, Tyner and Berry were proposed. On ballot Dr. Berry received the largest number of votes and was declared elected. Drs. J. W. Fennell, of Seguin, and P. W. Johns, of San Antonio, were unanimously elected First and Second Vice-President. For Secretary and Treasurer the names of Dr. E. J. Carothers and F. Terrell were proposed. On ballot Dr. Terrell was elected. Drs. Cupples, Tyner and King were elected censors. The retiring President, Dr. Watts, made a short address, reviewing the work completed during the past year, and spoke of the brilliant future which he thought was in store for the Association. The President appointed Drs. Cupples and Chew to escort the President-elect to the chair, and at the request of Dr. Tyner he, too, was placed on the committee. Dr. Berry, in a few remarks, thanked the Association for the high honor conferred upon him, coupled with the promise to perform the duties of the office to the best of his ability. The Association then adjourned to their annual banquet, which was served by that prince of caterers, Ludwig Mahncke, at the Mission Garden. Conviviality was the order of the evening, and all seemed to enter with zest into the enjoyment of the apetizing repast which "mine host" had so bountifully provided. Toasts were drunk, speeches made, songs sung, and the popping of corks from quart bottles of Mumm's extra dry kept up until the "we sma' hours" made their appearance. The tenth annual meeting and banquet of the West Texas Medical Association stands prominent among the many such happy occasions which have taken place in the annals of the society.

